# Fabrication of Porous Bone Scaffolds Using Alginate and Bioactive Glass

**DOI:** 10.3390/jfb10010015

**Published:** 2019-03-04

**Authors:** Jonathan Hatton, Graham Roy Davis, Abdel-Hamid I. Mourad, Nizamudeen Cherupurakal, Robert G. Hill, Sahar Mohsin

**Affiliations:** 1 Dental Physical Sciences Unit, Institute of Dentistry, Barts & The London School of Medicine and Dentistry, Queen Mary University of London, London E1 4NS, UK; jonathanrhatton@gmail.com (J.H.); g.r.davis@qmul.ac.uk (G.R.D.); r.hill@qmul.ac.uk (R.G.H.); 2 Department of Mechanical Engineering, College of Engineering, UAEU Al Ain 15551, UAE; ahmourad@uaeu.ac.ae (A.-H.I.M.); nizam.ac@uaeu.ac.ae (N.C.); 3 Department of Anatomy, College of Medicine and Health Sciences, United Arab Emirates University, Al Ain 17666, UAE

**Keywords:** bone scaffolds, alginate, bioactive glass, freeze-drying, porous, strontium

## Abstract

Porous composite scaffold using an alginate and bioactive glass ICIE16M was synthesized by a simple freeze-drying technique. The scaffold was characterized using compression testing, Fourier-transform infrared spectroscopy (FTIR), differential scanning calorimetry (DSC), X-ray diffraction (XRD), X-ray microtomography (XMT) and scanning electron microscopy (SEM). The bioactivity of the scaffold was evaluated by its ability to form apatite on its surface in simulated body fluid (SBF). The data collected showed evidence that the novel material produced had an appropriate pore size for osteoconduction, with an average pore size of 110 µm and maximum pore size of 309 µm. Statistical analysis confirmed that the glass filler significantly (*p* < 0.05) increased the collapse yield of the scaffolds compared with pure alginate scaffolds. The ICIE16M glass had an amorphous structure, favorable for bioactivity.

## 1. Introduction

Fractures impose a huge burden on healthcare systems. Autografts, allografts and metals have numerous limitations, including tissue availability/compatibility, pain, bleeding and potential for infection. There is a great need to develop synthetic bone graft substitutes to meet the epidemiologically driven demand. The ideal bone substitute should be biocompatible, porous, bioactive, bactericidal and mechanically stable. Interconnected porosity within the system is required to facilitate cell growth and vascularization.

Bone scaffolds are materials placed in an area of bone loss with the function of aiding hard tissue repair or supplementing bone tissue volume to facilitate the placement of an implant. The use of synthetic bone scaffolds is increasing due to the potential of these materials to alter the healing process. Additionally, they can be used for drug and growth factor delivery [1,2,3]. Although autografts and allografts remain the gold standard for bone scaffolds [4], various complications and morbidity [5,6,7,8] can be avoided with the use of synthetic bone scaffolds.

The use of bone substitutes is common in reconstructive surgery, including plastic surgery, orthopedics and, increasingly, in dentistry [9,10,11]. The economic impact of bone defects is huge, with an approximate annual cost of 2.5 billion dollars in the United States alone [12,13]. Furthermore, there are in excess of 2.2 million bone grafting procedures performed each year worldwide [14]. These figures can be expected to increase given the aging population, with an ever-increasing demand from patients for predictable outcomes following surgical interventions. 

Bone scaffolds provide a surface for bone cells to colonize and proliferate by providing mechanical stability and protection to the area and cell attachment sites [15]. Current literature [16,17,18,19] indicates that an average pore size of 100 µm in a bone scaffold is required in an interconnected arrangement for osteoprogenitor cell colonisation to allow new bone tissue to be formed. For larger defects, mature bone formation requires angiogenesis and invasion of nerve fibers. Much larger pores of 300 µm and upward are shown to be necessary for these processes [13,20,21]. However, an increased pore size may reduce cell adhesion due to a reduced volume to surface area ratio [15]. Porosity (ideally 90% of the volume of the scaffold) provides not only an increased surface area for cell migration, attachment and proliferation, but also for the diffusion of nutrients to the bone forming cells and for the passage of their waste products away from the local area [17]. Along with being adequately porous, an ideal bone scaffold should be biocompatible, bioactive, biodegradable, mechanically stable and reproducible for mass production. 

A variety of fabrication techniques are used in the bone tissue engineering field [22,23,24,25,26], such as particle leaching, sol-gel foaming, electrospinning [27,28,29], phase-separation, [30,31] freeze-drying, [32,33] and 3D printing [2]. Each technique has its advantages and disadvantages. For example, in the sol-gel foaming method, glass may degrade too rapidly before the bone can regenerate. The freeze-drying technique, however, is simple, does not require any expensive equipment and offers the benefit of being facilitating tailoring of the pore structure through variation of the lyophilisation process [21].

Alginate is polysaccharides obtained from certain species of brown algae and seaweed. It has long been used for many biomedical applications due to its biocompatibility, low toxicity and relatively low cost. It has been used as three-dimensional cell matrices to good effect, but it is now finding new applications in bone scaffold research [34,35,36]. Alginate can be easily shaped and manipulated. In addition, it is also a safe material and, hence, is commonly found in wound dressings such as Kalginate TM and as a filler material in the food industry. Alginate is a linear polymeric acid composed of 1, 4-linked β-d-mannuronic acid (M) and α-l-guluronic acid (G) residues. It forms hydrogel when certain divalent cations, such as Ca^2+^ or Sr^2+,^ are chemically crosslinked through ionic interaction between the cation and the carboxyl functional group of G units located on the polymer chain [18,20,21,37]. Alginates are shown to be able to support the nucleation of hydroxyapatite crystals on their surface when submerged in body fluids and SBF [38]. Previous work [20,21,35,36] showed the plausibility of using alginate to produce sufficiently porous bone scaffold materials. 

For several decades, the bioactive potential of ceramic-based materials in relation to bone formation was investigated, stemming from the work of Larry Hench [39], who developed the first such material, Bioglass^®^ 45S5. This work led to a snow-balling of interest in this field, giving rise to materials now routinely used to support bone healing, coat orthopedic implants and improve the interface between prostheses and living tissues [40]. A bioactive glass has been in use clinically as a synthetic bone void filler under the product name ‘Perioglas’ and ‘Novabone’ since 1993. Furthermore, such materials have the desired physical properties for use in various bone scaffold applications [41,42,43]. 

Bioactive silicate glasses are recognized as class ‘A’ bioactive materials because they offer high bioactivity involving both osteoconduction and osteoinduction. They are capable of reacting with the physiological fluids forming tenacious bonds to bone through the formation of bone-like hydroxyapatite layers leading to effective biological interaction and fixation of bone tissue with the material surface [5,6]. The bioactivity of glasses depends on their network connectivity (NC), which should be close to two [44]. 

Sriranganathan et al. [45] and Huang et al. [46] reported that Bioglass^®^ 45S5 has a slow degradation rate and HA formation rate when compared to other bioactive glasses, which makes it hard to predict the rate of new tissue formation in vivo. Moreover, it is difficult to induce porosity in scaffolds made from the Bioglass^®^. The main reason for this is that there is a small difference between its glass transition temperature and its crystallization temperature; the so-called processing or sintering window. These difficulties can lead to the formation of a scaffold that has a low strength/cohesivity [47]. For bioactive glasses to be effective as bone graft substitutes, they must be sintered into porous scaffolds without crystallization [46].

The first amorphous melt-derived bioactive glass scaffold suitable for bone applications was synthesized using the polymer foam replication technique with glass composition 13-93 [48]. This glass composition has a higher SiO_2_ content and low phosphorus content. Low phosphorus is associated with less bioactivity. Donnell et al. [49] reported that increasing the phosphorus content is associated with increased bioactivity. Secondly, it degrades at a rate that is even slower than Bioglass^®^ 45S5 [46]. However, an advantage that 13-93 has over Bioglass^®^ 45S5 is that it has a much larger sintering window, which means that porous 3D scaffolds can be created and sintered without crystallization. However, although the scaffolds made using the polymer foam replication technique [48,50] have significant porosity, they are very brittle [47].

The development of composite materials is common practice in tissue engineering and materials science. Composite materials can offer the beneficial properties of multiple materials. Indeed, bone tissue itself is a composite material of organic and inorganic phase [51].

In the light of the above, the objective of the study was to synthesize a novel, biocompatible composite material with sufficient porosity and with features suited to bone scaffold applications using a simple, reproducible freeze-drying technique. The composite is made by combining an alginate with strontium (Sr) and zinc (Zn) containing bioactive glass ICIE16M. The ICIE16M is modified from ICIE16 [52,53]. The glass ICIE16 has bioactivity comparable to 45S5, but large sintering window. In their study, Echezarreta-López et al. [52] showed the release of Sr and Zn ions from the ICIE16M glass. The presence of strontium and zinc in ICIE16M has a beneficial effect on bone growth [54,55]. Strontium and zinc both act by increasing bone formation and decreasing bone resorption, thus rebalancing bone turnover in favor of bone formation, an effect that results in increased bone mass and strength [56,57]. The inclusion of the glass filler is used to increase the strength of a pure alginate scaffold and promote bioactivity, which will promote in situ osseointegration [58,59].

## 2. Materials and Methods

### 2.1. Materials

Sodium alginate was obtained from Fisher Scientific Ltd (Leicestershire, UK), while calcium chloride and sodium lauryl sulphate were obtained from Sigma-Aldrich Ltd (Gillingham, UK). ICIE16M glass powder, which is a modification of ICIE16 (see Table 1), was synthesized as in previous protocols [52,53,54]. Compositions for ICIE16 and ICIE16M are given in Table 1. All the oxides were mixed together in the proportions as mentioned in Table 1 and heated to 1420 °C in a platinum crucible for 1.5 h, followed by quenching in water at room temperature [52,53,54]. The coarse frit form of the glass was collected and dried overnight. The glass frits were milled to powder form using a gyro-mill (Gyro mill, Glen Creston, London, UK) and sieved at <38 µm (Endecotts Ltd., London, UK). The glass particles were characterized by differential scanning calorimetry (DSC) and X-ray diffraction (XRD) techniques.

### 2.2. Methods

#### 2.2.1. Glass Characterization

The DSC for ICIE16M glass particles was conducted to determine the glass transition temperature (Tg) and crystallization temperature (Tc) [60,61,62,63,64]. A 50 µg sample of the glass powder was heated at rates of 10 °C min^−1^ and 20 °C min^−1^ using Stanton-Redcroft DSC 1500 (PL Thermal Sciences, Epsom, UK) series. A starting temperature of 50 °C was used and final temperature of 1100 °C using a Nitrogen atmosphere. 

X-ray diffraction spectrometry was performed on the samples (in powder form in silicone sample holder) using the PANalytical X'Pert Pro diffractometer (Malvern, UK) powered by Philips PW 1729 X-ray generator. Phase identification was carried out by means of the software program PANalytical High Score Plus (Version 2.2b, Malvern Panalytical Ltd, Malvern, UK). Diffraction data is acquired by exposing powder samples to Cu-Kα X-ray radiation, which has a characteristic wavelength (λ) of 1.5418 Å. X-rays were generated from a Cu anode supplied with 40 kV and a current of 40 mA.

#### 2.2.2. Fabrication of Composite Scaffold

One-hundred mL of deionized water was heated and maintained at a constant temperature of 50 °C in a 300 mL pyrex beaker. Three grams of alginate powder were added slowly along with 3 grams of ICIE16M glass powder and 100 mL of 0.2% sodium lauryl sulphate solution. The mixture was stirred at a constant rate of 2000 rpm for 20 min. The solution was poured immediately into 15 equal-sized cylindrical specimen tubes quenched in liquid nitrogen. After 5 min, the tubes were transferred into a commercial freezer at −20 °C. In the case of 15 control specimens, glass powder was not added in order to compare the mechanical properties of pure alginate scaffold with the composite scaffolds.

The samples were freeze-dried for 20 h at a pressure setting of 6 mbar and a temperature of −50 °C. These samples were then soaked in a 1% calcium chloride crosslinking solution for 3 h and thereafter refrozen in a commercial freezer at −20 °C for 48 h. The samples were then feeze-dried for a second cycle under the same conditions as before, resulting in a final composite bone scaffold being yielded. 

The density of the samples were calculated using the standard formulas:

Cross sectional area A = πr^2^ μm^2^ (1)

where r is the radius of the cylindrical sample

Volume of the sample (μm^3^) = Ah (2)

where h = the height (density = mass/volume g/μm^3^).

#### 2.2.3. Characterization of Scaffolds

Fourier-transform infrared spectroscopy (FTIR) technique was used as a quality control check to ensure that chemical crosslinking occurred within the body of the scaffold during fabrication using FTIR Spectrum GX with Spectrum v5.3.1 software (NICOLET IS10 FT-IR SPEC, Thermofisher, Waltham, MA, USA). FTIR provided detailed information on the chemical groups contained within the samples. Changes to the sample chemical structure can, in some instances, be shown by a shift in the original trace to different wave numbers. Solid samples of crosslinked and non-crosslinked scaffolds were tested between 4000 and 400 cm^−1^.

The specimens (1 mm × 2 mm × 3 mm) from composite scaffolds were prepared for X-ray microtomography (XMT). They were scanned using the in-house MuCAT 2 XMT scanner [65] with a 15 µm resolution. Image J (v1.43) and Drishti v2.4 software packages (developed by Ajay Limaye, Australian National University Supercomputer Facility, Australia) were used to assess XMT data. Average pore size calculations were completed using the ‘Thickness’ algorithm in the ‘Bone J’ plugin for Image J. 

Scanning electron microscopy (SEM) of composite scaffolds was conducted. Secondary electron beam images were taken up to 100×–800× magnification with SEM (JSM-840A, JEOL, Tokyo, Japan) to gain detailed information on scaffold architecture and the presence of any crystals following submersion of the scaffolds in SBF. 

Scaffolds were subjected to compression testing using an Instron 5567 universal testing machine (Instron Corp., Norwood, MA, USA). The samples were cut into cylinders of 8 mm diameter and 8 mm length with plane-parallel ends. The length and diameter were measured using a digital micrometer. Each sample was compressed with an overhead speed of 2 mm min^−1^ using a 1 kN load cell. The stiffness (Young’s Modulus E) of the scaffolds was obtained by fitting a tangent line to the initial part of the stress-strain plot. The gradient of this line is considered as the modulus of elasticity. A horizontal line was fitted against the plateau of the load versus time curve of the sample and the corresponding value of compressive load was taken for each specimen. The fracture stress was measured from the ‘load at collapse’ and cross-sectional area.

#### 2.2.4. Statistical Analysis

All pore sizes are expressed as mean ± standard deviation. Differences in collapse stress and Young’s modulus of glass reinforced and pure alginate scaffolds were assessed using the Student’s t-test, where a P value of less than 0.05 was considered significant.

#### 2.2.5. Evaluation of Bioactivity of Scaffolds in Simulated Body Fluid 

Reagents for preparation of simulated body fluid (SBF) solution [66]: Sodium chloride (NaCl), sodium hydrogen carbonate (NaHCO_3_), potassium chloride (KCl), di-potassium hydrogen phosphate trihydrate (K_2_HPO_4_.3H_2_O), magnesium chloride hexahydrate (MgCl_2_.6H_2_O), calcium chloride (CaCl_2_), sodium sulfate (Na_2_SO_4_), tris-hydroxymethyl aminomethane (TRIS) (HOCH_2_)_3_CNH_2_), 1M HCl solution. 

A solution of SBF was prepared according to the published method [66]. Composite scaffold samples were crushed in a ball shaker for exactly 1 min. The samples were weighed and the amount of SBF required for each sample was calculated according to the equation:

Volume of SBF needed = sample weight (g) × 50 mL/0.075 g

The specimens were placed in an agitator at 37 °C and the pH value was adjusted to 7.4 [67]. The solution was filtered at 8 h, 24 h, 120 h and 336 h (two weeks). The filtered scaffold material was washed with ethanol. SEM was performed on these samples to assess the formation of any crystals.

## 3. Results and Discussion

### 3.1. DSC Analysis of ICIE16M Glass Powder

**Figure 1:**
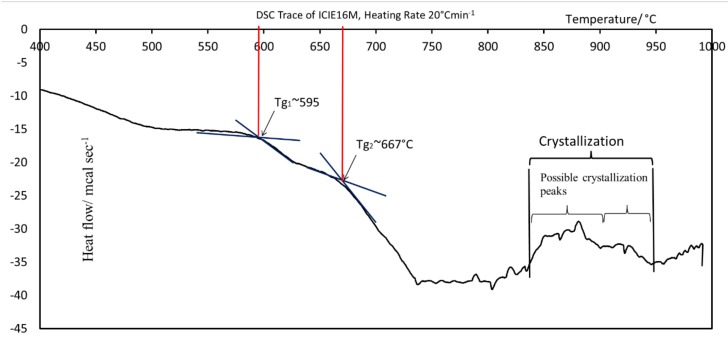
Differential scanning calorimetry (DSC) trace of ICIE16M glass powder at a heating rate of 10 °C min^−1^.

The trace from the DSC technique is shown in Figure 1. It presents the thermal changes occurring during the heating of a glass powder sample at a constant rate. Point A represents the first glass transition phase of the glass powder at 596 °C and point B represents the second glass transition temperature at 672 °C. ‘C’ represents the crystallization temperature range of the glass lying between 825 °C and 945 °C. It is worth mentioning here that the DSC test was conducted on five samples of glass ICIE16M. Figure 1 presents a representative curve. The deviation in the results was less than ±0.2%.

The sintering window was greater than 200 °C. The sintering window is defined as Tc − Tg, where Tc is the glass crystallization temperature and Tg is the glass transition temperature. The glass is most stable in a non-crystalline atomic arrangement. Amorphous atomic arrangements are believed to lead to the formation of an ‘open atomic network,’ which increases the in-situ reactivity of bioactive glasses. A stable and ordered crystalline atomic arrangement, by contrast, resists ionic dissolution in solution, so less reactive surface groups are made available for the nucleation of new crystals on their surface [68].

### 3.2. X-Ray Diffraction Analysis of ICIE16M Bioactive Glass

**Figure 2:**
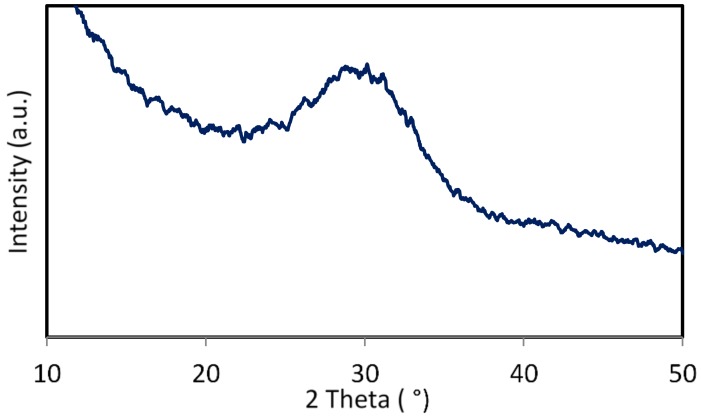
XRD trace of ICIE16M glass powder.

The curve obtained from XRD, as shown in Figure 2, confirms the lack of any significant crystal phases within the ICIE16M glass filler used in this work. Crystal phases are usually represented in the XRD trace by peaks and crystallization is known to reduce bioactivity [68,69]. In this study, XRD traces lacks any notable peaks. The ‘peaks’ observed in this graph are simply artefacts of the XRD process.

### 3.3. Fourier Transform Infrared Spectroscopy Analysis of Crosslinking

**Figure 3:**
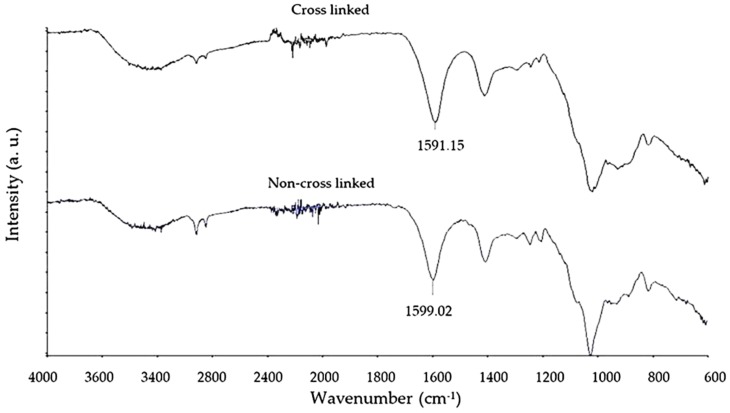
An overlay trace of FTIR data: Non-crosslinked and crosslinked scaffold samples in composite alginate and bioactive glass scaffold.

FTIR was used to compare the chemical composition of both non-crosslinked and crosslinked samples of the scaffold produced. The overlay FTIR trace in Figure 3 is concurrent with a previous study [16], indicating that the submersion of scaffolds in a crosslinking solution of calcium chloride solution for 3 h produced a notable chemical change in the scaffold and confirms crosslinking of the alginate polymer chains [16]. Characteristic portions of the trace are the hydroxyl bend at 3600 to 3000 cm^−1^ and the carbonyl (C=O) group represented by the peak (wave number labelled above) at approximately 1600 cm^−1^. There is an observed difference in wave number for the carbonyl group peak of approximately 9 cm^−1^, concurrent with the current literature [16].

### 3.4. X-Ray Microtomography and Scanning Electron Microscopy Imaging of Scaffolds

**Figure 4:**
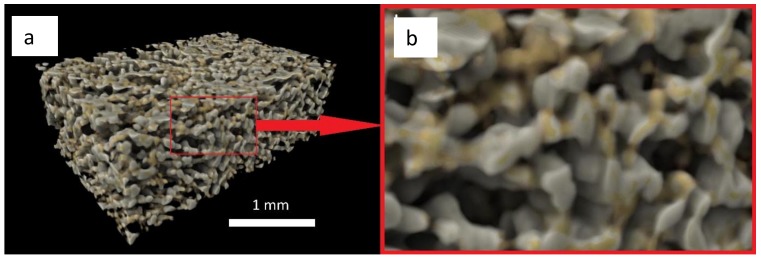
Computer rendered images taken using Drishti v2.4 of a composite scaffold showing (**a**) Porous structure of the scaffold in 3D and (**b**) zoomed section to better display the pore network (1 mm across).

**Figure 5:**
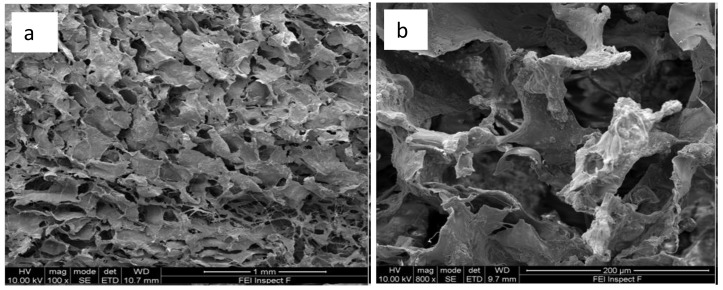
SEM Images of a composite scaffold. (**a**) Shows overall open pore architecture (100× magnification); (**b**) Shows the morphology of trabeculae (800× magnification).

X-ray microtomography (XMT) (Figure 4) and SEM (Figure 5) provide valuable information on the architecture of the pores contained within the scaffold material. The developed scaffolds were analyzed in 3D using the Drishti v2.4 software (developed by Ajay Limaye, Australian National University Supercomputer Facility, Australia) that allowed investigating qualitatively the distribution and interconnectivity of the pores within the sample. The 3D computer rendered images show that the composite scaffold exhibits an extensive, interconnected porous network throughout its structure Figure 4a,b. Computer analysis of the XMT stack data allowed for quantification of the pore sizes contained within the scaffold. Average pore sizes within the produced scaffolds were calculated to be 109.8 µm ± 39.8 µm (Table 2), therefore falling within the intended range. The high-resolution images gained from SEM, as shown in Figure 5a,b, provide a greater understanding of the pores on the surface of the scaffolds produced. The image taken at higher magnification (Figure 5b) shows in details the morphology of the trabeculae within the composite scaffold. Current literature [70] indicates that osteoprogenitor cell colonization and proliferation is only made possible when average pore sizes of 100 µm are present to allow for cell movement (osteoblasts for example are approximately 20–25 µm in size) and, importantly, the diffusion of nutrients to these cells and the movement of their waste products away from the area [17,70].

Interconnectivity of the pores allows for bone formation at the centre of the defect. Studies using nonporous scaffolds show promising bone formation at the peripheries of the defect only [70,71]. The maximum pore size obtained of 308.9 µm (Table 2) is also within the recommended range and reflects the natural architecture of trabecular bone. These larger pores are essential for mature bone formation in larger bone defects as they facilitate the permeation of blood vessels and nerve fibres.

### 3.5. Compression Testing of Scaffolds

**Figure 6:**
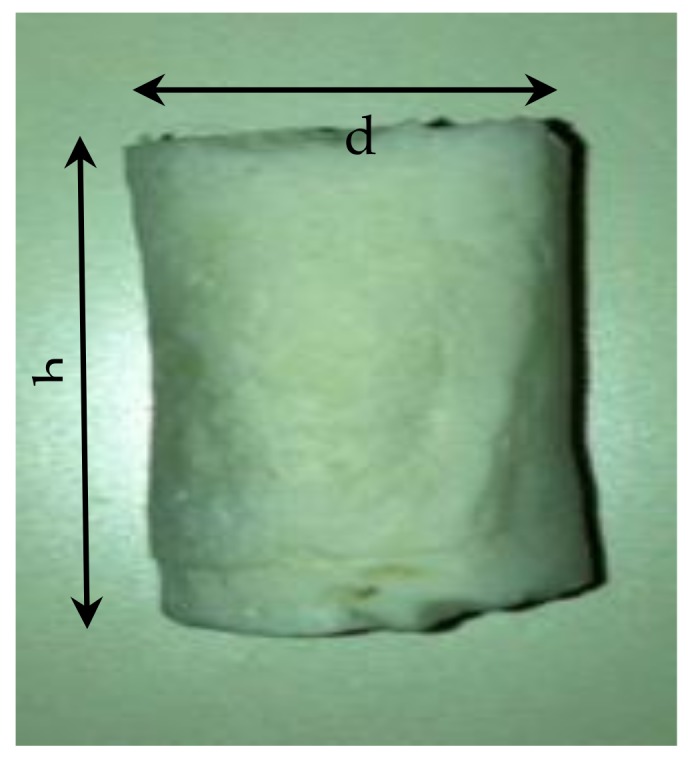
Composite scaffold after production for compression testing.

**Figure 7:**
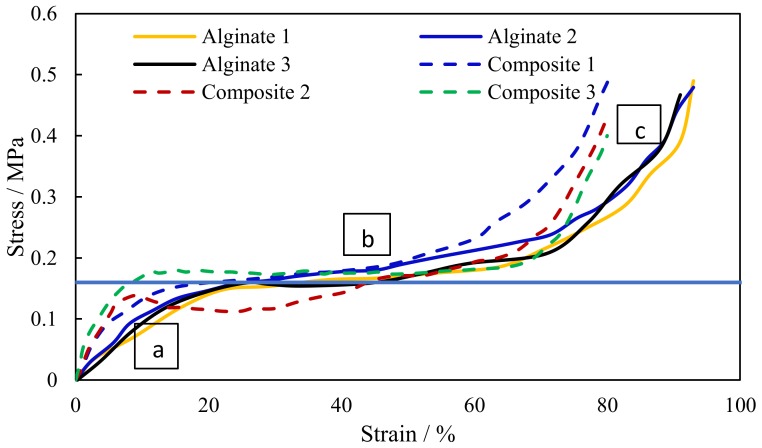
Representative stress-strain curves for pure alginate and composite scaffolds. (**a**) Elastic deformation region; (**b**) Plastic deformation region; (**c**) Densification region.

Figure 6 shows the fabricated composite scaffold ready for compression testing. Compression tests were conducted for both pure alginate and composite samples. From the compression stress-strain curve (Figure 7) of the scaffolds, the Young’s Modulus and collapse stress were obtained. As with all cellular solids, the developed scaffolds exhibit three characteristic phases of deformation under load. An initial linear trace labelled ‘A’ is seen where trabeculae within the body of the scaffold are deforming elastically up to the first peak (point of zero slope). Removal of the load in the range of this linear extension would result in the scaffold returning to its’ original form with no permanent damage. The plateau of the graph (Figure 7) labelled ‘B’ is the result of plastic deformation of the trabeculae when they are loaded beyond their collapse stress. Further loading of the specimen beyond this limit causes densification of the trabeculae [72], so an increase in the gradient of the curve is seen at Figure 7 labelled ‘C’. The tests have been conducted on 15 samples for each material (pure alginate and composite scaffolds). Figure 7 presents only three representative curves for each to visualize the repeatability of the results. From the plot, the point at which the curve tends to a plateau can be considered as the yield/collapse strength of the sample. The modulus of elasticity is calculated from the slope of the initial part of the stress-strain curve (Figure 7). 

The data obtained from the compression testing (Table 3 and Figure 7) show that the inclusion of bioactive glass particle filler significantly increases (*p* = 0.02) the collapse stress/yield stress of the composite scaffolds (0.21 MPa) when compared with pure alginate scaffolds (0.16 MPa) produced in the same manner. The effect on modulus, however, was not significant (*p* ˃ 0.05). More studies are underway to further improve the mechanical properties by changing the composition of glass and by adding different proportions of alginate to bioglass. 

### 3.6. Bioactivity of Composite Scaffolds

**Figure 8:**
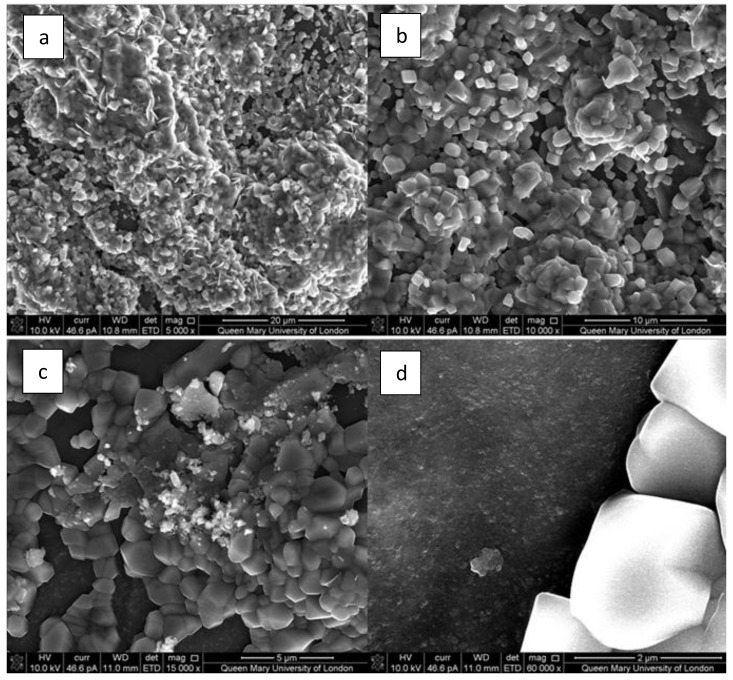
SEM of composite scaffold submerged in SBF for two weeks. (**a**) 5000×, (**b**) 10000×, (**c**) 15000×, (**d**) 60000×.

The composite scaffolds were assessed for bioactivity. An abundance of crystallites are seen to cover the surface of composite bone scaffold, as seen in the SEM image (Figure 8). Some specimens exhibited crystal growth after 120 h of submersion in SBF (Figure 6a–c), but for most samples, crystals appeared after two weeks, as shown in an earlier study [54,73]. The morphology of crystals also does not seem to be concurrent with that expected of hydroxyapatite crystals. These crystals exhibited cuboidal morphology (Figure 8c,d) of calcite formations. 

ICIE16 is also known to form calcium phosphate crystals on its surface, which is a known precursor to hydroxyapatite after only 8 h submersion in SBF. After three days, typical needle-like hydroxyapatite crystals appear [53]. It could be that strontium substitution for calcium in ICIE16M bioactive glass has inhibited apatite-like phase formation. Moreover, bonding with alginate may have affected the bioactivity. The network connectivity of ICIE16M (calculated to be 2.13) may not be correct and Zn^2+^ and Mg^2+^ were probably acting more as network formers and NC may be close to 2.53 [54,73]. Strontium and zinc present in ICIE16M are necessary for several enzymatic processes within the body. Strontium in the form of strontium ranelate is prescribed for its antiresorptive and anabolic properties to osteoporotic patients [74]. Previous studies [54,73] showed the release of strontium and zinc ions in SBF from ICIE16M using dissolution studies. 

## 4. Conclusions

The ability to synthesize porous, biocompatible novel composite scaffold using alginate and bioactive glass (ICIE16M) containing strontium and zinc was demonstrated. Zinc and strontium are anabolic to bone and additionally zinc has antibacterial activity. The ICIE16M glass had an amorphous structure confirmed through XRD, and hence was favorable for bioactivity. The synthesized crosslinked and non-crosslinked scaffold materials were characterized using FTIR and compared with the available literatures. Adequate porosity of the scaffold material was confirmed by SEM and XMT techniques. However, more experiments are required to improve the bioactivity and mechanical strength of these scaffolds. The results obtained are useful and can funnel future research into experimentation with different bioactive glass fillers using larger crosslinking cations, such as strontium and barium, to improve the mechanical integrity of the alginates. Additionally, the bioactivity of the composite could be further enhanced by inclusion of nano-hydroxyapatite particles. Such material will provide a wide range of potential applications in the area of biomedical engineering.

## Figures and Tables

**Table 1:** Compositions (mol. %) of alginate and bioactive glass (ICIE16M) and ICIE16 [52,53,54].

	SiO_2_	Na_2_O	CaO	SrO	K_2_O	MgO	ZnO	P_2_O_5_
ICIE16M	49.46	6.60	27.27	3.00	6.60	3.00	3.00	1.07
ICIE16	49.46	6.60	36.27	0	6.60	0	0	1.07

**Table 2:** Computer analysis of data obtained for composites scaffolds using XMT and SEM techniques.

Mean Trabecular Thickness (µm)	Max. Trabecular Thickness (µm)	Average Density (g µm^−3^)
41.715 ± 11.37	108.165	9.63 × 10^−14^
Mean pore Size (µm)	Max. pore Size (µm)	SA to weight ratio (m^2^g^−1^)
109.8 ± 39.81	308.865	0.005

**Table 3:** Average Young’s Modulus and Collapse stress for pure alginate and composite scaffolds.

Sample Type	Average^*^ Young’s Modulus (MPa)	*p*-Value	Average Collapse Stress/Yield Stress (MPa)	*p*-Value
Alginate Scaffold	1.82 ± 0.99	0.9	0.159 ± 0.01	0.022
Composite Scaffold	1.83 ± 0.66		0.175 ± 0.04	
